# Analysis of survivin, XIAP and FoxM1 as potential chemoresistance factors in breast cancer cells

**DOI:** 10.1186/1753-6561-7-S2-P27

**Published:** 2013-04-04

**Authors:** Gabriela N Moraes, Flavia C Vasconcelos, Deborah Delbue, Karina L Silva, Giuliana P Mognol, João P Viola, Raquel C Maia

**Affiliations:** 1 Program of Molecular Hematology-Oncology, National Cancer Institute, Rio de Janeiro, Brazil; 2 Program of Cellular Biology, National Cancer Institute, Rio de Janeiro, Brazil

## Background

Survivin and XIAP inhibitor of apoptosis proteins (IAPs) and FoxM1 transcription factor overexpression are associated with poor prognosis in breast cancer. This work aimed at investigating the role of these proteins in doxorubicin (dox) resistance in breast cancer cells.

## Materials and methods

Human breast carcinoma cell lines MCF7 (wild-type p53) and MDA-MB-231 (mutant p53) were exposed to dox and citotoxicity was assessed through the MTT assay. Apoptosis was detected through analysis of flow cytometry DNA content and caspases-3, -7 and -9 levels. Survivin, XIAP, p53 and FoxM1 levels were assessed by Western blotting and Survivin and XIAP mRNA levels, by Real Time PCR. Transfections with the Survivin-encoding plasmid and siRNA against Survivin and XIAP were performed using Lipofectamine reagent.

## Results

Dox induced DNA fragmentation and reduction in procaspases-3, -7 and -9 levels in both cells, independently of their p53 status. Upon dox-mediated apoptosis, Survivin and XIAP levels were reduced. Survivin-induced overexpression did not confer cells a dox resistant-phenotype and Survivin silencing, alone or in combination with XIAP, did not result in enhanced sensitivity to the drug. Furthermore, Survivin and XIAP mRNA levels were downregulated after dox treatment, concurrently with FoxM1 transcription factor inhibition.

## Conclusion(s)

Our data show that Survivin and XIAP downregulation are not crucial events in dox-induced apoptosis and suggest that their regulation might be a consequence of FoxM1 transcription factor inhibition. It still remains to be investigated whether FoxM1 modulation directly affects IAPs expression and cell sensitivity to dox-mediated apoptosis.

## Financial support

FINEP, INCT and Ministério da Saúde.

